# The critical effects of self-management strategies on predicting cancer survivors’ future quality of life and health status using machine learning techniques

**DOI:** 10.1371/journal.pone.0330570

**Published:** 2025-08-28

**Authors:** Ju Youn Jung, Young Ho Yun

**Affiliations:** 1 Division of Tourism & Wellness, Hankuk University of Foreign Studies, Yongin, Republic of Korea; 2 Department of Family Medicine, Seoul National University College of Medicine, Seoul, Republic of Korea; 3 Department of Human System Medicine, Seoul National University College of Medicine, Seoul, Republic of Korea; 4 Medical Big Data Research Center, Medical Research Center, Seoul National University, Seoul, Republic of Korea; Athens Medical Group, Psychiko Clinic, GREECE

## Abstract

Despite the significance of enhancing the quality of life (QoL) and overall health status (including physical, mental, social, and spiritual well-being) among individuals who have survived cancer, the existing prediction model for QoL and health status lacks sufficient interpretation. Our primary objectives were to develop and validate simple prediction models for QoL and secondary health statuses. Additionally, we aimed to interpret these prediction models using explainable artificial intelligence (XAI) methods, including extracting important features and creating dependence plots. Lastly, we sought to predict and interpret individual outcomes, visualizing the results using the XAI technique known as SHapley Additive explanation (SHAP). In this prospective cohort study, conducted through a web-based survey, we established prediction models for QoL and health statuses, comparing their performance with ensemble methods, including decision trees, random forest, gradient boosting, eXtreme Gradient Boost (XGBoost), and LightGBM. Following the model comparison, we selected the XGBoost model for further analysis. We identified crucial features associated with QoL and each health status separately and leveraged SHAP to extract individual prediction results from the XGBoost model. After preprocessing the data and selecting the appropriate model, our final dataset consisted of 256 cancer survivors with 42 predictive features. Repeated stratified K-fold validation demonstrated high performance of the XGBoost predictive model for QoL. Similarly, the XGBoost predictive model exhibited good performance for each health status, including mental, social, and spiritual well-being. The important features identified in these predictive models varied based on the specific health outcomes. This study represents the first endeavor to develop and validate predictive models for QoL and health status among cancer survivors while also providing interpretations of these models.

## Introduction

Cancer survivors are continuously increasing in South Korea [1]. In South Korea, the recent 5-year cancer survival rate was 72.9% (2018–2022). Globally, this trend is also accelerating, with the total number of individuals diagnosed with cancer projected to rise significantly over the next several decades due to population ageing and growth. If current incidence trends continue, the global cancer burden is expected to approximately double by 2070 compared to 2020 [2].

The increase in the number of cancer survivors has also increased the interest in improving their quality of life (QoL) and various health statuses such as physical, emotional, social, and spiritual health statuses because they often suffer from various physical, psychological, social, and spiritual difficulties [3–8]. Cancer requires continuous management like a chronic disease due to the continuation of various difficulties accompanying cancer as well as the management of the disease itself [9]. Thus, self-management strategies are necessary for cancer survivors to take care of their own health to improve QoL and physical, psychological, social, and spiritual health statuses.

The Smart Management Strategies for Health Assessment Tool (SAT) has been recently developed to help cancer survivors practice self-management continuously and overcome various difficulties that come with cancer [10]. The strategies outlined by the SAT aim to overcome health crises and facilitate positive growth that can be applied in a time-specific manner according to the treatment period and health behavior stages of cancer survivors [10]. The SAT includes the following 15 sub-strategies in three sets, namely, SAT-C (Core), SAT-P (Preparation), and SAT-I (Implementation) [10]. A previous study reported that the more SAT strategy clusters used by cancer survivors, the better their QoL and overall health status (physical, mental, social, and spiritual) [11]. However, since that study clustered SAT strategies, it could not examine which strategies were important features individually predicting the QoL or each health status.

Recent advancements in machine learning (ML) have led to an increasing number of studies predicting clinical outcomes in cancer patients, with some models demonstrating superior performance compared to traditional statistical approaches [12]. In particular, models with simple and interpretable structures are gaining attention for their potential application in real-world clinical settings [13]. For example, eXtreme Gradient Boosting (XGBoost) based models have achieved high accuracy in predicting survival among patients with metastatic cancer, and the use of explainable AI techniques such as SHAP (Shapley Additive Explanations) has allowed for clear identification and visualization of important predictive features, thereby supporting the design of personalized interventions [13]. However, despite the clinical relevance of quality of life (QoL) as a strong predictor of survival, studies using ML to predict QoL among cancer survivors remain scarce [14–16]. Developing an accurate QoL prediction model could enable early identification of patients at risk for poor QoL and facilitate timely, tailored interventions. Such models have the potential to improve long-term survivorship care by offering data-driven insights into individual needs and guiding customized health management strategies.

Therefore, in this study, we intend to develop and validate a QoL and health status prediction model for cancer survivors that can be easily applied in actual clinical settings to improve the survivors’ QoL. To facilitate actual clinical applications, this study attempted to simply organize the input variables of the machine learning prediction model into sociodemographic and clinical variables and SAT strategies for cancer survivors. Thus, this study identified the following three detailed research objectives with the ultimate goal of providing customized healthcare services for cancer survivors to improve their QoL and health status: (1) Development and validation of models to predict QoL primarily and overall health statuses (physical, mental, social, and spiritual) secondarily for cancer survivors; (2) explaining the prediction of the above model using explainable artificial intelligence (XAI) techniques and extracting important features that predict QoL and overall health statuses; and (3) predicting the QoL and overall health statuses of individual cancer survivors using XAI techniques and visualizing them to suggest a method for providing customized healthcare services.

## Methods

### Study design

In this study, we used *HealthingU*, a digital health assessment platform, to collect multidimensional health data from participants at baseline and after 6 months. The platform enables structured evaluation of physical, mental, and behavioral health factors, and provides tailored feedback to promote self-management and behavior change (S1 Fig in S1 File). Participants were encouraged to complete the surveys on the website, but those who found it difficult to take an online survey received the survey as well as the results of the survey by mail. This study was conducted after approval by the institutional review board (IRB) of Seoul National University Hospital. The reference number was 1308-087-514. Data were collected between 01-09-2013 and 31-08-2014. The study design and survey instruments have been previously detailed [11].

### Study participants

We included cancer survivors who met the following inclusion criteria: 1) diagnosed with cancer, 2) over 18 years of age, 3) able to read and write Korean, 4) able to use the Internet and email, and 5) submitted written informed consent. A total of 540 cancer survivors participated in this study after submitting informed consent and completing a web-based survey with a coordinator’s support in outpatient clinics from four hospitals in South Korea. Among these 540 cancer survivors, 256 cancer survivors who completed the second survey after 6 months were ultimately included in this study. This study was conducted after obtaining approval from the institutional review boards of the four hospitals. All methods used were conducted in compliance with applicable guidelines and regulations.

### Measurements

Our questionnaire included questions regarding sociodemographic and clinical variables (age, sex, marital status, residence, income, education, religion, cancer stage, cancer type, and treatment stage), the SAT for self-management strategies [10,11], the European Organization for Research and Treatment of Cancer (EORTC) QoL questionnaire 30 (EORTC-QLQ-C30) for using the global QoL [17], and the Health Status Questionnaire (HSQ), which evaluated physical, mental, social, and spiritual health statuses [18,19]. Detailed information of measurements was described in Table 1–3 [20].

**Table 1:** Sociodemographic and clinical characteristics of the study participants.

Features	Category	N (%) or Mean ± SD	Min-Max
Age		51.28 ± 9.64	23–77
Sex	Male	91 (35.5)	
	Female	165 (64.5)	
Income (won)	<4,000,000	125 (48.8)	
	≥4,000,000	131 (51.2)	
Education	≤High school graduates	97 (37.9)	
	≥University graduates	159 (62.1)	
Residence	Other areas	102 (39.8)	
	Metropolitan area	154 (60.2)	
Marriage	Single	37 (14.5)	
	Married	219 (85.5)	
Religion	No	94 (36.7)	
	Yes	162 (63.3)	
Cancer type	Breast cancer	93 (36.3)	
	Lung cancer	69 (27)	
	Colon cancer	57 (22.3)	
	Gastric cancer	17 (6.6)	
	etc.	20 (7.8)	
Cancer stage	I, II	118 (46.1)	
	III, IV	138 (53.9)	
Treatment stage	≤5 years after treatment	86 (33.6)	
	>5 years after treatment	170 (66.4)	

SD, standard deviation

**Table 2:** Features of the smart management Strategies for Health Assessment Tool (SAT) included in the dataset.

Features	Mean ± SD	Min-Max
**Baseline**		
**SAT-C**		
Proactive problem-solving strategy	64.1 ± 18.93	10–100
Positive-reframing strategy	68.42 ± 21.44	14.8–100
Creating empowered relationship strategy	77.06 ± 18.73	11.1–100
Experience-sharing strategy	48.78 ± 25.19	0–100
**SAT-P**		
Goal and action setting	48.68 ± 21.19	0–100
Rational decision-making strategy	63.54 ± 17.74	22.2–100
Healthy environment-creating strategy	57.32 ± 19.81	0–100
Priority-based planning strategy	54.69 ± 21.22	0–100
Life value-pursuing strategy	61.12 ± 20.68	6.7–100
**SAT-I**		
Self-motivating strategy	57.63 ± 19.49	4.8–100
Self-implementing strategy	54.13 ± 21.58	0–100
Reflecting strategy	44.79 ± 27.16	0–100
Energy-conserving strategy	58.42 ± 19.27	0–100
Activity-coping strategy	57.58 ± 22.70	0–100
**Change**		
**SAT-C**		
Proactive problem-solving strategy	−1.18 ± 16.84	−100–36.7
Positive-reframing strategy	−1.35 ± 18.03	−91.6–51.9
Creating empowered relationship strategy	−4.32 ± 17.38	−94.4–38.9
Experience-sharing strategy	−1.74 ± 24.28	−88.9–66.7
**SAT-P**		
Goal and action setting	0.13 ± 19.62	−90–53.3
Rational decision-making strategy	−3.08 ± 17.10	−88.9–55.6
Healthy environment-creating strategy	−0.62 ± 18.09	−60–46.7
Priority-based planning strategy	−1.04 ± 19.83	−75–58.3
Life value-pursuing strategy	−3.33 ± 18.82	−73.3–46.7
**SAT-I**		
Self-motivating strategy	−1.10 ± 17.99	−76.2–61.9
Self-implementing strategy	−0.42 ± 19.79	−75–58.3
Reflecting strategy	−3.32 ± 27.98	−100−100
Energy-conserving strategy	−0.95 ± 20.78	−77.8–20.78
Activity-coping strategy	−0.31 ± 21.38	−86.7–73.3

SD, standard deviation

**Table 3:** Descriptive summary of outcome features in the dataset.

Features	Category	N (%)
**Primary outcome**		
Global Quality of Life	High	227 (88.67)
	Low	29 (11.33)
**Secondary outcomes**		
Physical health status	Good	170 (66.41)
	Bad	86 (33.59)
Mental health status	Good	210 (82.03)
	Bad	46 (17.97)
Social health status	Good	227 (88.67)
	Bad	29 (11.33)
Spiritual health status	Good	193 (75.39)
	Bad	63 (24.61)

### Data preprocessing

Prior to model development, several preprocessing procedures were performed.

First, missing data were imputed using the Markov Chain Monte Carlo (MCMC) multiple imputation algorithm, which accommodates arbitrary missing patterns and handles both categorical and continuous variables simultaneously [21].

Second, categorical variables were converted into binary features for machine learning analyses (Tables 1–3), except for “cancer type,” which was encoded using the one-hot encoding method. Continuous variables were retained in their original scale to support personalized healthcare recommendations based on score magnitudes [20].

Third, the primary outcome, global QoL, was binarized to enhance clinical interpretability. According to established thresholds, a score of 33.33 or below was considered clinically problematic [22–24]. The secondary outcome was categorized as “Good” (1 = Excellent, 2 = Very Good, 3 = Good) or “Bad” (4 = Bad, 5 = Very Bad).

Lastly, to mitigate multicollinearity, one variable—specifically, a self-sustaining strategy from the SAT—was removed due to high correlation with other features (Pearson r > 0.7) [25,26]. Although multicollinearity does not impair model prediction accuracy, it may distort interpretation of variable importance. Therefore, we excluded the variable to improve the explanatory reliability of the model [20].

All preprocessing was conducted using SAS OnDemand for Academics 2025 version (SAS Institute Inc., Cary, NC, USA).

### Machine learning techniques

Various machine learning algorithms were used to create predictive models and compare their performance. In this study, we only used ensemble methods such as decision trees, random forest, gradient boosting, XGBoost, and LightGBM. These ensemble methods did not require preprocessing, such as scaling and normalization of each feature. We selected ensemble methods to use the scores of survivors’ survey results without any processing for developing use in clinical settings based on the collected patient-reported outcome (PRO) values from patients, enabling direct application in clinical practice. We finally selected the XGBoost algorithm to develop predictive models and extract feature importance and individual prediction results with using Shapley Additive Explanation (SHAP) values.

### Model development and evaluation

To develop a predictive model for global quality of life (QoL) and health statuses among cancer survivors, we trained an XGBoost classifier using a simple set of features, including sociodemographic information, clinical characteristics, and self-management assessment scores (SAT). All analyses were implemented in Python 3.8 using open-source libraries such as scikit-learn and xgboost (version 0.81).

Hyperparameter optimization was conducted using GridSearchCV to identify the optimal learning rate and number of trees (n_estimators), while other parameters (e.g., max_depth, gamma, subsample) were fixed based on prior studies to minimize overfitting and improve model stability.

To benchmark the performance of the XGBoost model, we additionally trained and evaluated four widely used ensemble-based classifiers: decision tree, random forest, gradient boosting, and LightGBM. These models were trained on the same dataset and evaluated using identical procedures to ensure fair comparison.

**Fig 1:**
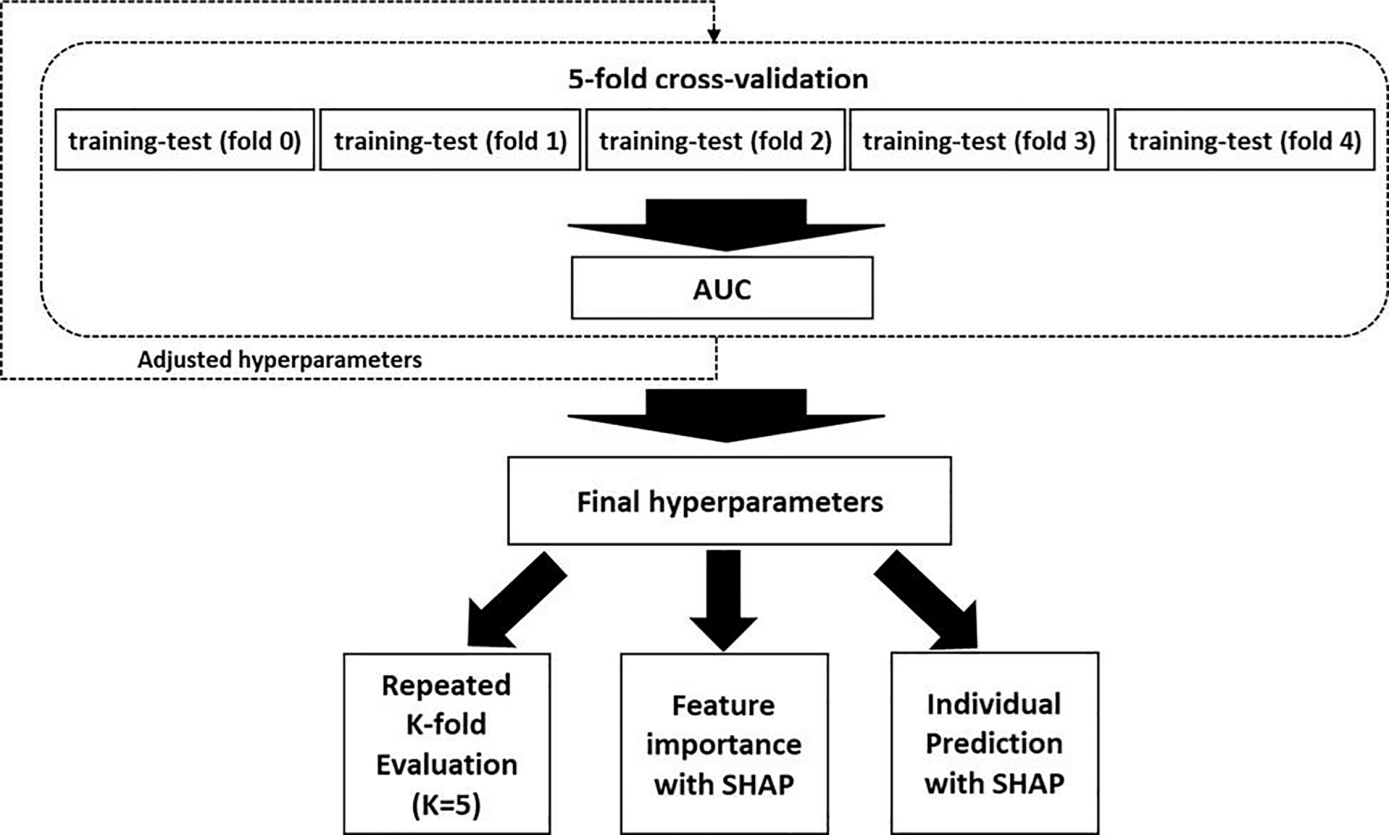
Overview of model development, evaluation, feature interpretation, and individual prediction.

To assess predictive performance, we calculated AUROC, AUPRC, accuracy, and F1-score. We also applied repeated stratified K-fold cross-validation (K = 5, repeats = 20) to ensure robustness and mitigate the impact of class imbalance (Fig 1) [20–22].

To enhance explainability, SHAP (SHapley Additive exPlanations) values were computed to interpret feature contributions at both the global and individual levels. Visualizations were generated using the shap package (version 0.29.1).

## Results

### Study participants and features

A total of 256 cancer survivors were included in the dataset after preprocessing. Table 1 shows the mean or prevalence of sociodemographic and clinical features. The mean SAT baseline and change scores are described in Table 2.

Table 3 shows the outcome features in the dataset, with 88.67% showing a high global QoL (primary outcome), 66.41% showing good physical health status, 82.03% showing good mental health status, 88.67% showing good social health status, and 75.39% showing good spiritual health status (secondary outcome).

### Model performance

Table 4 compared XGBoost and other ensemble classifiers for the primary outcome (global QoL) [20]. XGBoost produced the best results for the main performance measures, i.e., the AUROC and AUPRC. Accuracy and F1 scores of XGBoost yielded the second-best results. Assessment of the prediction performance of XGBoost showed an AUROC of 0.80 (95% CI, 0.78 to 0.82), AUPRC of 0.96 (95% CI, 0.95 to 0.97), and F1 scores of 0.88 (95% CI, 0.84 to 0.92) and 0.93 (95% CI, 0.91 to 0.95). The final tuned hyperparameters are described in S2 Table in S1 File.

**Table 4:** Comparison of predictive performance across methods for primary outcome.

Methods	AUROC (mean, 95% CI)	AUPRC (mean, 95% CI)	Accuracy (mean, 95% CI)	F1 score (mean, 95% CI)
Decision Tree	0.63 (0.61–0.65)	0.91 (0.90–0.92)	0.88 (0.83–0.90)	0.94 (0.92–0.95)
Random Forest	0.78 (0.76–0.80)	0.96 (0.95–0.97)	0.88 (0.87–0.90)	0.94 (0.93–0.95)
Gradient Boosting	0.78 (0.76–0.80)	0.95 (0.94–0.96)	0.87 (0.81–0.92)	0.93 (0.89–0.95)
XGBoost	0.80 (0.78–0.82)	0.96 (0.95–0.97)	0.88 (0.84–0.92)	0.93 (0.91–0.95)
LightGBM	0.79 (0.77–0.81)	0.96 (0.95–0.97)	0.89 (0.88–0.90)	0.94 (0.94–0.95)

AUROC, area under the receiver operating characteristic curve; AUPRC, area under the precision–recall curve; CI, confidential interval.

Table 5 shows the prediction performance results for health statuses (secondary outcome) using XGBoost [20]. Overall, the predictive performance for the secondary outcome was slightly lower than that for the primary outcome. The predictive results for physical health status included an AUROC of 0.66 (95% CI, 0.65 to 0.67), AUPRC of 0.77 (95% CI, 0.75 to 0.79), accuracy of 0.69 (95% CI, 0.57 to 0.78), and F1 score of 0.79 (95% CI, 0.72 to 0.84). The predictive results for mental health status included an AUROC of 0.71 (95% CI, 0.69 to 0.73), AUPRC of 0.90 (95% CI, 0.89 to 0.92), accuracy of 0.83 (95% CI, 0.78–0.86), and F1 score of 0.90 (95% CI, 0.88 to 0.93). The predictive results for social health status included an AUROC of 0.77 (95% CI, 0.75–0.79), AUPRC of 0.96 (95% CI, 0.95–0.97), accuracy of 0.88 (95% CI, 0.84 to 0.92), and F1 score of 0.94 (95% CI, 0.92 to 0.96). The predictive results for spiritual health status included an AUROC of 0.75 (95% CI, 0.74 to 0.76), AUPRC of 0.87 (95% CI, 0.85 to 0.89), accuracy of 0.79 (95% CI, 0.71 to 0.88), and F1 score of 0.87 (95% CI, 0.81 to 0.92).

**Table 5:** Predictive performance of the xgboost model for health status outcomes.

**Outcomes**	**AUROC (mean, 95% CI)**	**AUPRC (mean, 95% CI)**	**Accuracy (mean, 95% CI)**	**F1 score (mean, 95% CI)**
Physical Health Status	0.66 (0.65–0.67)	0.77 (0.75–0.79)	0.69 (0.57–0.78)	0.79 (0.72–0.84)
Mental Health Status	0.71 (0.69–0.73)	0.90 (0.89–0.92)	0.83 (0.78–0.86)	0.90 (0.88–0.93)
Social Health Status	0.77 (0.75–0.79)	0.96 (0.95–0.97)	0.88 (0.84–0.92)	0.94 (0.92–0.96)
Spiritual Health Status	0.75 (0.74–0.76)	0.87 (0.85–0.89)	0.79 (0.71–0.88)	0.87 (0.81–0.92)

AUROC, area under the receiver operating characteristic curve; AUPRC, area under the precision-recall curve; CI, confidential interval.

The prediction performance of AUROC and AUPRC are depicted in S3–S5 Figs in S1 File. S4 Fig in S1 File shows the AUROC and AUPRC for predicting the primary outcome (global QoL) in the XGBoost model, and S4 Fig in S1 File shows the AUROC and AUPRC of different algorithms for predicting the global QoL to compare the performance results with XGBoost. S5 Fig in S1 File shows the AUROC and AUPRC for predicting the secondary outcomes (overall health statuses) in the XGBoost models.

### Feature importance and individual prediction

We applied the final tuned XGBoost models to extract SHAP values in each primary and secondary outcome predictive model. S6–S7 Figs in S1 File showed the feature importance bar plots and the beeswarm plots for each model’s top ten important features. The top three important features for predicting the global QoL were the activity-coping strategy, the proactive problem-solving strategy, and the change values of the self-implementing strategy for six months. For predicting the physical health status, the top three important features were the activity-coping strategy, the healthy environment-creating strategy, and age. For predicting mental health status, the top three important features were proactive problem-solving, positive reframing, and rational decision-making strategies. For predicting social health status, the top three features were activity-coping, healthy environment-creating, and self-motivating strategies. For predicting spiritual health status, the top three features were the positive-reframing strategy, religion, and income. Thus, the top three most important features for each predictive outcome were different. The baseline SAT scores were found to be more important than the change scores of the SAT strategy for six months in predicting outcomes.

We selected two samples using the global QoL model and examined the compositions of individual predictions. In S8 Fig in S1 File (A), the predicted global QoL was 2.07, suggesting that the global QoL was high (true) for this survivor. However, the survivor was living alone, and the low baseline score of the rational decision-making strategy in SAT-P had a negative influence (blue arrows) on the QoL. In S8 Fig(B) in S1 File, the predicted QoL was −2.58, suggesting this survivor’s QoL was low (false). Although there are some positive effects on the QoL (red arrows), such as no reduction in using the self-implementing strategy for six months, the low baseline score of the proactive problem-solving strategy in SAT-C, the activity-coping strategy in SAT-I, the self-motivating strategy in SAT-I, and the reduction in using the activity-coping strategy for six months had more negative effects on the global QoL. As a result, this survivor’s global QoL turned out to be low (false)

## Discussion

In this study, we developed and validated various models for predicting cancer survivors’ QoL primarily and overall health statuses such as physical, mental, social, and spiritual health statuses secondarily. The QoL of cancer survivors was highly predictable only with basic personal characteristics such as sociodemographic and clinical variables and the scores of self-management strategies (SAT strategies) (AUROC = 0.80, AUPRC = 0.96). The development of a simple model for predicting QoL can increase the ease of application to improve the QoL of cancer survivors in clinical settings.

The most important feature predicting the QoL of cancer survivors was the activity copying strategy of the SAT-I. Previous studies have described the direct and indirect influence of the activity copying strategy. Popa-Velea et al. reported that the activity coping strategy significantly influences QoL through resilience [23], and other studies have shown that coping strategies are directly beneficial to the QoL and psychosocial adaptation of cancer survivors [24,25]. In this study, among the 15 strategies of SAT, the activity-coping strategy was found to have the most significant influence on the QoL of cancer survivors. Thus, active usage of coping strategies can be recommended for cancer survivors to improve their QoL.

The second important variable was the proactive problem-solving strategy of SAT-C [10]. As mentioned in the background of this study, cancer survivors experience various physical, mental, social, and spiritual difficulties more than the general population [3–7]. Therefore, the use of the proactive problem-solving strategy to actively address these problems could have a positive effect on the QoL. The third important variable was the change value of the self-implementing strategy in SAT-I. This is an indicator of the usage of the self-implementing strategy by cancer survivors for six months, and usage of this strategy more than the baseline was significant in predicting the QoL. The self-implementing strategy is a strategy for implementing the health behaviors and treatment behaviors necessary for health determined by cancer survivors [10]. The use of the self-implementing strategy may affect health behaviors, which might be beneficial to QoL

The predictive performance of health statuses was modestly lower than that of the QoL in cancer survivors. Except for the physical health status predictive models, the models for mental, social, and spiritual health statuses showed good performance: all AUROCs were over 0.70, and all AUPRCs were above 0.87. In a previous study, when SAT strategies were clustered, and physical health status was compared between the groups with high and low strategy scores, the group with the high strategy score also showed a high average physical health status score. However, this study showed that the performance of SAT scores in classifying the goodness of physical health status was not very good. Perceived health status and QoL are distinct constructs [26]. Although physical, mental, social, and spiritual health status constitutes a part of the quality of life, the predictive model for SAT strategies and personal characteristics predicted QoL better than all health statuses. The QoL has a multi-dimensional nature, combining various factors such as physical, psychological, and social health statuses and subjective evaluation of environmental facets surrounding human beings [27]. Interestingly, our simple model with SAT scores predicted the QoL determined by these complex factors well. In addition, important features for the QoL and each health status appeared differently, suggesting that a customized self-management strategy considering each survivor’s QoL or health status is necessary when providing healthcare services for cancer survivors.

In this study, the enhanced iteration of Gradient Boosting, known as the XGBoost model, exhibited increased accuracy and robustness, achieving an AUC of 0.80. It could be because the SAT strategy, already known for its high correlation with QoL, was utilized as a key variable to predict QoL. XGBoost demonstrated robust predictive capabilities even with a small sample size. To further evaluate the model’s calibration, we calculated the Brier Score, which yielded a value of **0.07**, indicating that the model’s predicted probabilities were well-calibrated and aligned closely with actual outcomes. A Brier Score closer to 0 reflects better accuracy in probabilistic predictions, and this result supports the reliability of the model [28]. The importance matrix of the XGBoost model reflects the variables’ contribution to predicting quality of life, implying that higher-ranked variables are more useful in predicting a better quality of life. These outcomes can serve as crucial evidence for interventions to enhance cancer survivors’ quality of life.

This study attempted to develop a web-based questionnaire targeting cancer survivors. Our team hoped this approach would provide helpful health information to cancer survivors while allowing research. To achieve that goal, the *HealthingU* website was developed, which allowed survivors to view personalized survey results about their health as soon as they completed the web-based survey. In this study, we found a way to provide customized healthcare information using SHAP, a newly developed XAI technology [29,30]. SHAP’s individual prediction is a new function that is unavailable in existing traditional statistical methods, and this technology will open up a new horizon in providing customized healthcare services for cancer survivors. We derive insights for personalized healthcare plans from SHAP’s individual prediction outcomes. This approach, as seen in S8 Fig in S1 File, allows tailoring health improvement strategies to each survivor based on factors impacting their quality of life. For instance, for the survivor (A) in S8 Fig in S1 File, emphasizing the red-marked strategies is crucial while intervening more with the blue-coded Rational decision-making strategy. The survivor (B) requires focused education on low-scoring yet impactful blue strategies. This personalized approach aims to enhance patients’ quality of life and overall health outcomes.

In predictive models, clinical variables such as survival have predominantly been used as dependent factors. In contrast, variables like quality of life have been valued solely as major influencing factors affecting clinical outcomes. However, quality of life variables hold significant value as they can be modified through intervention, unlike clinical variables. Hence, we proposed self-management strategies as novel influencing variables affecting quality of life. This study, considering both demographic, clinical characteristics, and self-management strategies, could provide evidence that the quality of life prognostic indicators, primarily the strategies utilized in this study (SAT strategies), could be pivotal intervention elements for enhancing the quality of life of cancer survivors even within clinical settings. Algorithms designed for individual assessment of cancer survivors’ quality of life could steer clinical decision-making systems, providing enhanced insights into personalized care through predictive models. These novel findings regarding quality of life enhancement present a tangible avenue for specific interventions.

This study had several limitations. First, the number of participants in this study was relatively small, and an independent external test cohort was not available. Due to the nature of the prospective cohort design and the limited availability of comparable external data, we could not conduct external validation. However, this study involved multicenter recruitment from four independent hospitals in Korea, and we collected 6-month follow-up data directly through the HealthingU program. To mitigate potential overfitting and to assess model generalizability in the absence of external validation, we conducted hyperparameter tuning and applied repeated stratified K-fold validation (K = 5, repeats = 20) [31,32]. In addition, we also evaluated the XGBoost model using the bootstrap validation method (n_iteration = 300) [22]. The prediction performance after the bootstrap validation (AUROC = 0.77, AUPRC = 0.97) was similar to the predictive result of the stratified repeated K-fold validation (S9 Fig in S1 File). Despite these efforts to ensure robustness, the relatively small sample size remains a limitation. Therefore, it is necessary to recruit a larger number of research participants in future studies to confirm if similar results can be consistently replicated, thereby strengthening the generalizability and reliability of our findings. Second, the completion rate for the second survey completion rate was approximately 47% of the completion rate for the first survey. The first survey was conducted in outpatient clinics with a coordinator’s support. The second survey was answered online by the survivor alone, while cancer survivors who did not respond to the survey online within the period completed the survey by mail. In the second survey, most survivors completed the survey by mail. Since the average age of the participants in this study was 51.3 years old, it would have been difficult for older people to complete the web-based questionnaire alone. Support seems necessary to increase the web-based survey completion rate of the elderly. In addition, the user interface and user experience of the web-based survey may have to be customized to increase the ease of its use for the elderly. Third, SHAP can provide individual prediction results. However, the interpretation of the statistical significance of SHAP values was impossible in the context of current ML models due to the absence of an established system [33]. Future studies should specifically aim to accumulate data examining whether the individual predictions of SHAP have statistically significant positive effects on cancer survivors’ quality of life or health status, rigorously evaluated through verifiable research methods like randomized controlled trials (RCTs). If the effectiveness of individual prediction results of SHAP can be verified through several clinical studies, this approach will be more beneficial for cancer survivors in clinical practice. Fourth, while our model demonstrated promising predictive capabilities, we acknowledge that incorporating more clinical variables could further enhance its performance and generalizability. Our current model primarily focused on variables readily available through the HealthingU program and routinely collected clinical data, ensuring practical applicability and ease of data collection in a real-world setting. However, future research should explore integrating additional comprehensive clinical markers or patient-reported outcomes to offer a more nuanced understanding and potentially improve model performance.

This study first developed and validated simple prediction models of QoL primarily and health status secondarily that were strongly associated with cancer survivorship. We also produced important features predicting the health outcomes and individual predictions for cancer survivors and interpreted the results. These study results can be used effectively for promoting cancer survivors’ QoL and health status and developing customized healthcare plans in clinical settings.

## Supporting information

S1 File S1 Fig. Representative Samples: HealthingU Web-Based Survey and Patient Report. S2 Table. XGBoost Model’s Optimized Hyperparameters for Global QoL Prediction. S3 Fig. XGBoost Model Performance for Global QoL: (A) AUROC and (B) AUPRC. S4 Fig. Comparative Performance of Different Algorithms for Global QoL Prediction (AUROC and AUPRC). S5 Fig. XGBoost Model Performance for Health Statuses: AUROC and AUPRC. S6 Fig. Feature Importance for Global QoL Prediction by the XGBoost Model: Beeswarm and Bar Plots. S7 Fig. Feature Importance for Overall Health Status Prediction by the XGBoost Model: Beeswarm and Bar Plots. S8 Fig. Individual Patient Sample: (A) Positive and (B) Negative Global QoL Compositions from SHAP Predictions. S9 Fig. XGBoost Model Performance for Global QoL Prediction After Bootstrap Validation: AUROC and AUPRC.
(ZIP)
