# Supplementary figures and images for: The critical effects of self-management strategies on predicting cancer survivors’ future quality of life and health status using machine learning techniques
Source: PLoS One. 2025 Aug 28;20(8):e0330570. doi: 10.1371/journal.pone.0330570 (PMC12393778; doi:10.1371/journal.pone.0330570)

S3 Fig. XGBoost Model Performance for Global QoL: (A) AUROC and (B) AUPRC

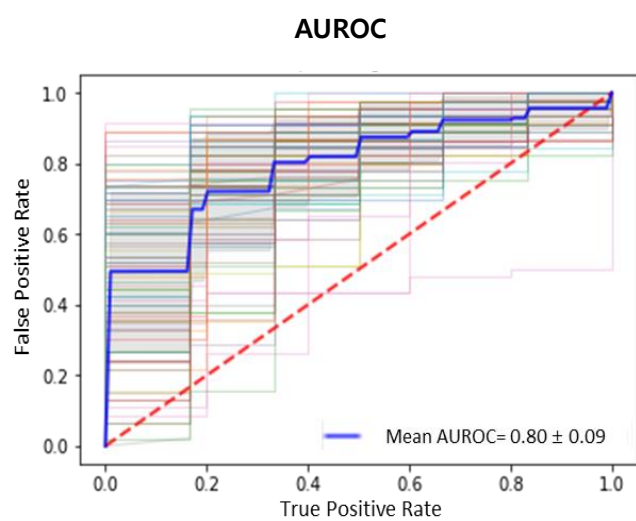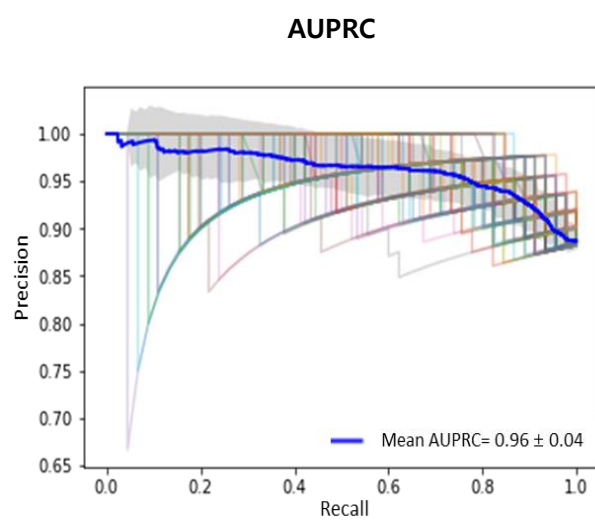

Supplement: S1 File — S1 Fig. Representative Samples: HealthingU Web-Based Survey and Patient Report. S2 Table. XGBoost Model’s Optimized Hyperparameters for Global QoL Prediction. S3 Fig. XGBoost Model Performance for Global QoL: (A) AUROC and (B) AUPRC. S4 Fig. Comparative Performance of Different Algorithms for Global QoL Prediction (AUROC and AUPRC). S5 Fig. XGBoost Model Performance for Health Statuses: AUROC and AUPRC. S6 Fig. Feature Importance for Global QoL Prediction by the XGBoost Model: Beeswarm and Bar Plots. S7 Fig. Feature Importance for Overall Health Status Prediction by the XGBoost Model: Beeswarm and Bar Plots. S8 Fig. Individual Patient Sample: (A) Positive and (B) Negative Global QoL Compositions from SHAP Predictions. S9 Fig. XGBoost Model Performance for Global QoL Prediction After Bootstrap Validation: AUROC and AUPRC. (ZIP) [file pone.0330570.s001.zip › Supporting Information 1_9/Supporting Information 3.pdf]

S5 Fig. XGBoost Model Performance for Health Statuses: AUROC and AUPRC

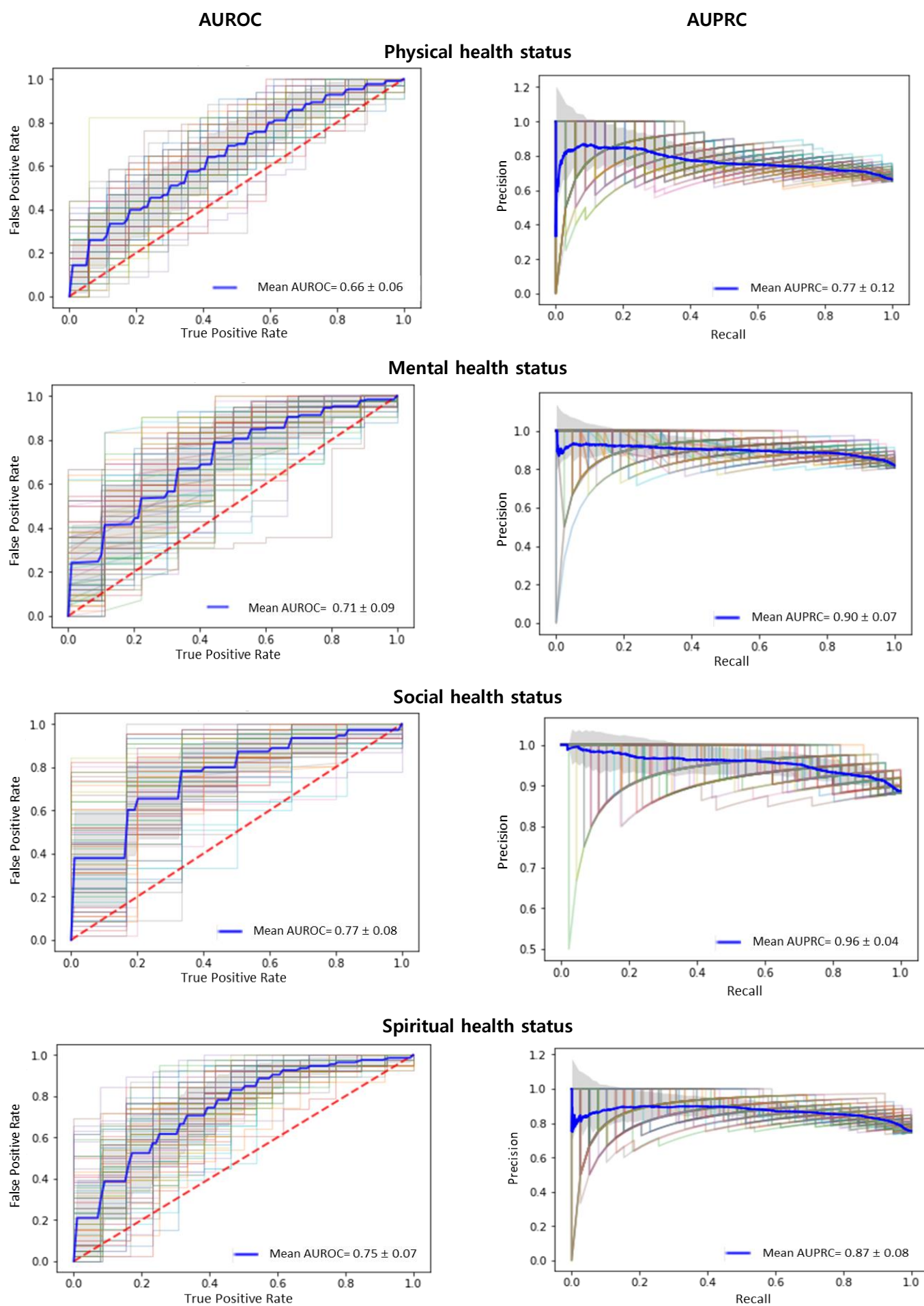

Supplement: S1 File — S1 Fig. Representative Samples: HealthingU Web-Based Survey and Patient Report. S2 Table. XGBoost Model’s Optimized Hyperparameters for Global QoL Prediction. S3 Fig. XGBoost Model Performance for Global QoL: (A) AUROC and (B) AUPRC. S4 Fig. Comparative Performance of Different Algorithms for Global QoL Prediction (AUROC and AUPRC). S5 Fig. XGBoost Model Performance for Health Statuses: AUROC and AUPRC. S6 Fig. Feature Importance for Global QoL Prediction by the XGBoost Model: Beeswarm and Bar Plots. S7 Fig. Feature Importance for Overall Health Status Prediction by the XGBoost Model: Beeswarm and Bar Plots. S8 Fig. Individual Patient Sample: (A) Positive and (B) Negative Global QoL Compositions from SHAP Predictions. S9 Fig. XGBoost Model Performance for Global QoL Prediction After Bootstrap Validation: AUROC and AUPRC. (ZIP) [file pone.0330570.s001.zip › Supporting Information 1_9/Supporting Information 5.pdf]

S6 Fig. Feature Importance for Global QoL Prediction by the XGBoost Model: Beeswarm and Bar Plots

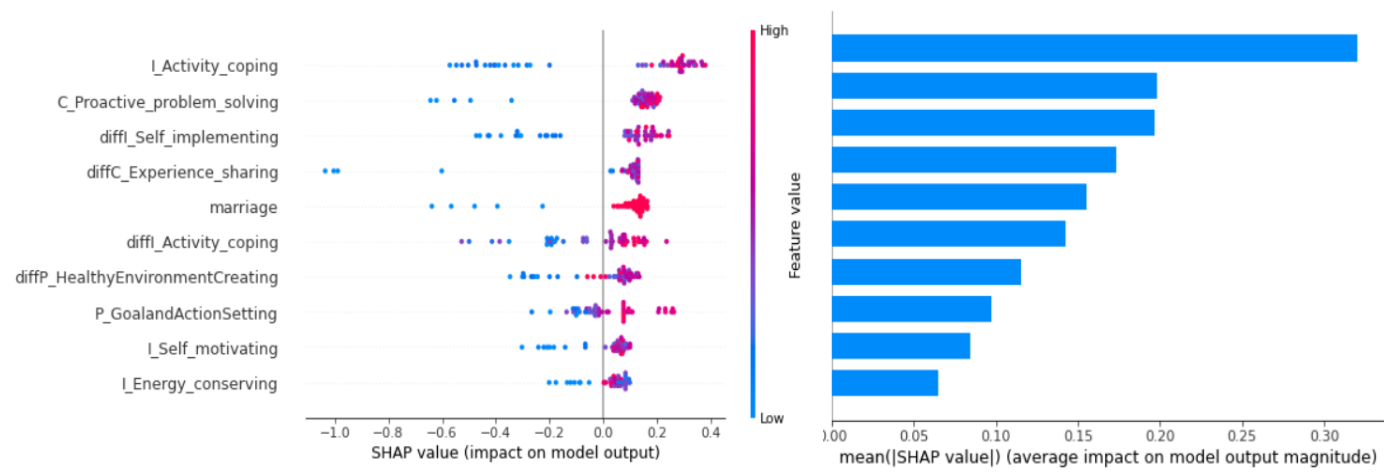

Supplement: S1 File — S1 Fig. Representative Samples: HealthingU Web-Based Survey and Patient Report. S2 Table. XGBoost Model’s Optimized Hyperparameters for Global QoL Prediction. S3 Fig. XGBoost Model Performance for Global QoL: (A) AUROC and (B) AUPRC. S4 Fig. Comparative Performance of Different Algorithms for Global QoL Prediction (AUROC and AUPRC). S5 Fig. XGBoost Model Performance for Health Statuses: AUROC and AUPRC. S6 Fig. Feature Importance for Global QoL Prediction by the XGBoost Model: Beeswarm and Bar Plots. S7 Fig. Feature Importance for Overall Health Status Prediction by the XGBoost Model: Beeswarm and Bar Plots. S8 Fig. Individual Patient Sample: (A) Positive and (B) Negative Global QoL Compositions from SHAP Predictions. S9 Fig. XGBoost Model Performance for Global QoL Prediction After Bootstrap Validation: AUROC and AUPRC. (ZIP) [file pone.0330570.s001.zip › Supporting Information 1_9/Supporting Information 6.pdf]
